# Evaluation of monocyte indices as predictors of adalimumab response in patients with psoriasis: A retrospective real-world data study

**DOI:** 10.1016/j.jdin.2026.08.001

**Published:** 2026-08-15

**Authors:** Ole Krogh-Jensen, Nikolai Loft, Diljit Kaur-Knudsen, Claus Zachariae, Lone Skov, Amanda Kvist-Hansen

**Affiliations:** a Department of Dermatology and Allergy, Copenhagen University Hospital – Herlev and Gentofte, Hellerup, Denmark; b Department of Clinical Medicine, Faculty of Health and Medical Sciences, University of Copenhagen, Copenhagen, Denmark; c Leo Foundation Skin Immunology Research Center, Department of Immunology and Microbiology, Faculty of Health and Medical Sciences, University of Copenhagen, Copenhagen, Denmark

**Keywords:** adalimumab, biologic therapy, monocyte indices, psoriasis, response prediction

*To the Editor:* Biomarkers for predicting treatment response in psoriasis remain limited and identifying patients with psoriasis who will achieve and maintain a clinical response to adalimumab is a key challenge. To date, no clinically validated biomarkers specifically predicting response to adalimumab in patients with psoriasis exist. Circulating monocyte levels are readily available inflammatory measures from routine blood tests and a recent study associated high monocyte levels with a suboptimal response to adalimumab in patients with hidradenitis suppurativa (HS).^1^ Monocytes have also been associated with adalimumab response in rheumatoid arthritis (RA).^2^ However, little is known about their utility in psoriasis. Therefore, we investigated whether monocyte-based indices were associated with response to adalimumab in patients with moderate-to-severe psoriasis.

We conducted a retrospective cohort study using real-world data from adult patients with psoriasis treated with adalimumab at the Department of Dermatology and Allergy, Herlev and Gentofte Hospital from October 2015 to March 2023. We included data from patients where a psoriasis area and severity index (PASI) could be matched with blood monocyte and lymphocyte counts at 1 or more predefined time points (baseline, 3 and 12 months). Patients achieving a PASI≤2 at 3 or 12 months were categorized as responders.^3^ Baseline monocyte count, and monocyte-to-lymphocyte ratio (MLR) were evaluated as predictors of adalimumab response using logistic regression and receiver operating characteristic (ROC) curve analyses (Table I). Cross-sectional comparisons between responders and non-responders at each follow-up were assessed using Wilcoxon rank-sum test. (Figure 1).

**Table I:** Patient characteristics and results of multivariable logistic regression and receiver operating characteristic (ROC) curve analyses across study timepoints

Patient characteristics			
Variable	Baseline (*n* = 166)	Month 3 (*n* = 122)	Month 12 (*n* = 102)
Sex, no. (%), male	110 (66.3)	74 (60.7)	69 (67.6)
Age, mean (SD), y	45.4 (15.4)	46.3 (14.7)	46.1 (14.2)
Weight∗, mean (SD), kg	89.0 (21.9)	86.2 (19.4)	88.8 (19.7)
PASI, median (IQR)	8.9 (5.5-12.5)	1.2 (0.0-2.6)	1.0 (0.0-2.5)
Monocyte count 10^9^/L, median (IQR)	0.40 (0.30-0.50)	0.40 (0.32-0.50)	0.40 (0.31-0.52)
MLR, median (IQR)	0.23 (0.18-0.31)	0.19 (0.15-0.23)	0.18 (0.14-0.23)
Biologic Naïve, no. (%)	141 (84.9)	104 (85.2)	86 (84.3)
Results			
MLR, unadjusted OR per 0.1 increase (95% CI)	-	1.33 (0.90-1.99)	1.04 (0.67-1.64)
P value for MLR, unadjusted	-	0.155	0.850
MLR, adjusted OR per 0.1 increase (95% CI)	-	1.17 (0.73-1.88)	0.92 (0.54-1.55)
P value for MLR, adjusted	-	0.507	0.0744
Monocyte count, unadjusted OR per 0.1 × 10^9^/L increase (95% CI)	-	1.22 (0.89-1.66)	1.00 (0.72-1.40)
P value for Monocyte count, unadjusted	-	0.211	0.984
Monocyte count, adjusted OR per 0.1 × 10^9^/L increase (95% CI)	-	1.24 (0.87-1.77)	0.85 (0.57-1.27)
P value for Monocyte count, adjusted	-	0.233	0.427
ROC for MLR, Area under curve (95% CI)	-	0.54 (0.41-0.66)	0.50 (0.35-0.64)
ROC for Monocyte count, Area under curve (95% CI)	-	0.55 (0.43-0.67)	0.50 (0.36-0.64)

Values are shown as mean ± standard deviation (SD) or median (interquartile range [IQR]). Categorical variables are presented as number (%). Odds ratios (OR) are reported for non-response, which is defined as a psoriasis area and severity index (PASI) greater than 2. The monocyte-to-lymphocyte ratio (MLR) OR is reported per 0.1 increase and the monocyte count OR per 0.1 × 10^9^/L increase. Adjusted variables include age, weight, baseline PASI and biologic naivety.

∗ Missing for n = 6 at baseline, n = 3 at month 3, and n = 4 at month 12.

**Fig 1:**
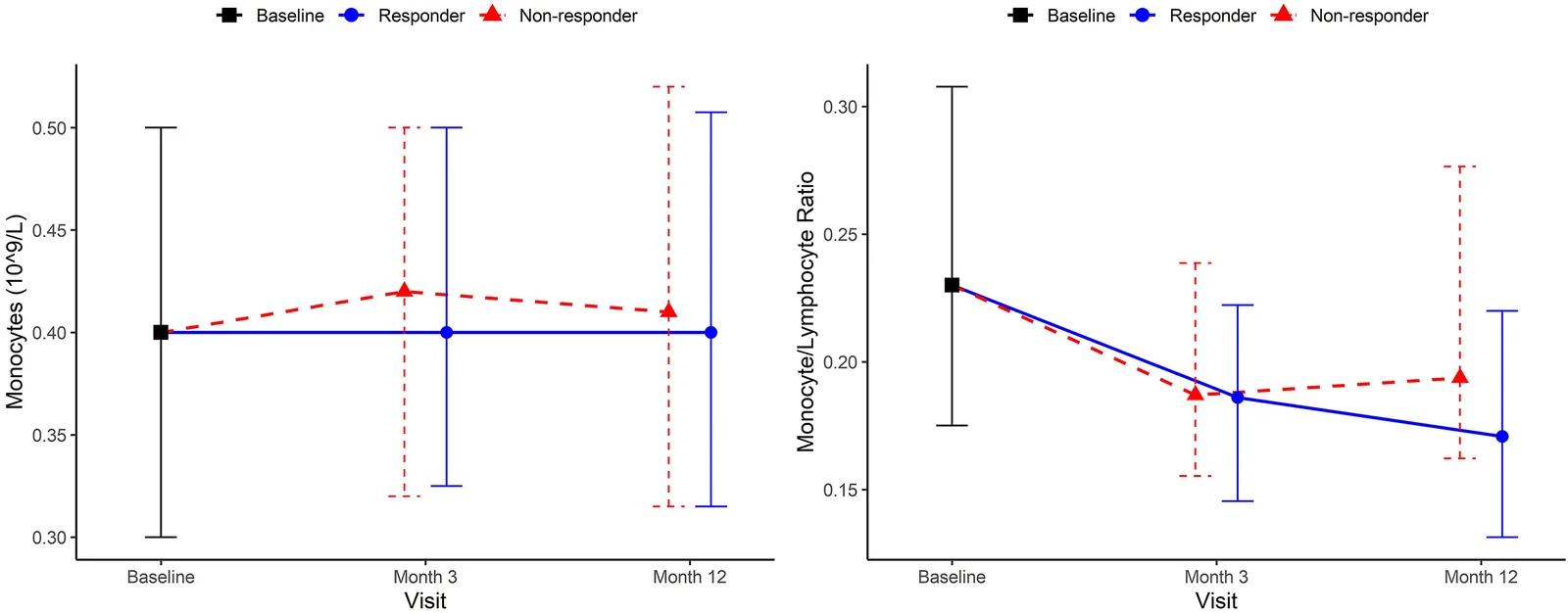
Monocyte indices during adalimumab treatment in patients with psoriasis. Monocyte count and monocyte-to-lymphocyte ratio at baseline, 3 months, and 12 months in adalimumab treated patients with psoriasis. Follow-up visits are stratified by treatment response. The analysis included 166 patients at baseline, 122 patients at 3 months (79 responders vs 43 non-responders), and 102 patients at 12 months (70 responders vs 32 non-responders).

The study included 166 patients at baseline, 122 and 102 patients at 3 and 12 months, respectively (Table I). Median monocyte count was 0.4 10^9^/L at all timepoints. MLR levels were slightly lower at the 3- and 12-month treatment time points than at baseline. Neither monocyte count nor MLR showed an association with treatment response at 3 or 12 months using multivariable logistic regression (Table I). Model estimates were similar with and without adjustment for potential confounders. ROC analyses showed poor discrimination of baseline monocyte count and MLR for prediction of clinical response at both 3 and 12 months (Table I). No significant differences in monocyte counts or MLR were observed between responders and non-responders 3 months (*P* = 0.59 and *P* = 0.52, respectively) or 12 months (*P* = 0.52 and *P* = 0.097, respectively) (Figure 1). A sensitive analysis where patients who discontinued their treatment due to lack of efficacy before the defined time points were included in the non-response group did not change the results.

Contrary to findings in RA and HS,^1^^,^^2^ monocyte counts and MLR do not seem to predict adalimumab response in patients with psoriasis. Monocyte levels remained constant at all investigated time points. MLR levels where slightly lower at the later time points, probably reflecting changes in lymphocyte counts, rather than monocytes counts. In line with this, Andersen et al reported that low baseline neutrophil-to-lymphocyte ratio (NLR) was associated with response to adalimumab in patients with psoriasis.^4^ Similarly, Morariu et al found limited predictive value of monocyte-based indices for treatment response in psoriasis, whereas NLR and systemic inflammatory response index were predictors of being a super responder.^5^ Our findings do not support circulating monocyte levels as a biomarker for adalimumab response in psoriasis.

### Declaration of generative AI and AI-assisted technologies in the writing process

Not applicable.

## Conflicts of Interest

O Krogh-Jensen, D Kaur-Knudsen and A Kvist-Hansen declares no conflict of interest. N Loft reports research grant funding from the Leo Foundation and that he has been a paid speaker for Eli Lilly, Janssen Cilag, Galderma, and Sandoz. L Skov has been a paid speaker for AbbVie, Eli Lilly, Takeda, Sanofi, Pfizer and LEO Pharma, and has been a consultant or has served on Advisory Boards with AbbVie, Janssen Cilag, Eli Lilly, LEO Pharma, UCB, Almirall, Bristol-Myers Squibb, Takeda, Stada and Sanofi and has received research and educational grants from Sanofi, Bristol-Myers Squibb, UCB, Almirall and Janssen-Cilag. C Zachariae has been a paid speaker for Eli Lilly, Novartis, CSL, UCB and LEO Pharma and has been a consultant or has served on Advisory Boards with Abb Vie, Janssen Cilag, Novartis, Eli Lilly, LEO Pharma, UCB, Almirall, Takeda, Amgen and CSL.
